# Immunotherapy in Recurrent Ovarian Cancer

**DOI:** 10.3390/biomedicines13010168

**Published:** 2025-01-12

**Authors:** Keyao Chen, Jingjing Wang, Meng Yang, Shaoqiong Deng, Li Sun

**Affiliations:** Gynecology Department, National Cancer Center/National Clinical Research Center for Cancer/Cancer Hospital & Shenzhen Hospital, Chinese Academy of Medical Sciences and Peking Union Medical College, Shenzhen 518116, China; m18294456609@163.com (K.C.); wjj01947@163.com (J.W.); yangmeng6364@163.com (M.Y.); dengshaoqiong112@163.com (S.D.)

**Keywords:** recurrent ovarian cancer, immunotherapy, immune microenvironment

## Abstract

Background/Objectives: It remains challenging to treat recurrent ovarian cancer effectively as traditional interventions like chemotherapy and surgery have limited long-term efficacy, highlighting an urgent need for innovative approaches. Immunotherapy offers potential advantages in modulating the immune response against tumor cells and has emerged as a promising strategy in ovarian cancer management. This review discusses various immunotherapy modalities, including active and passive immune strategies, for recurrent ovarian cancer. Methods: We systematically reviewed recent immunotherapy advances for recurrent ovarian cancer, including the efficacy and mechanisms of single and dual immune checkpoint inhibitors, checkpoint inhibitor combinations with chemotherapy or radiotherapy, anti-angiogenic agents, PARP inhibitors, antibody–drug conjugates (ADC), tumor vaccines, and adoptive cell therapies (ACT). Additionally, we assessed emerging research on biomarkers predictive of immunotherapy responsiveness in ovarian cancer. Results: The findings indicate that immunotherapy, particularly combinations involving immune checkpoint inhibitors and other agents, demonstrates promising efficacy in recurrent ovarian cancer, with some therapies showing enhanced benefits in specific subtypes. The immune microenvironment in platinum-sensitive and -resistant cases exhibits distinct immunological profiles, influencing therapy outcomes. Several potential biomarkers have been identified, potentially aiding in patient stratification and treatment optimization. Conclusions: Immunotherapy significantly advances recurrent ovarian cancer treatment, with various combinations potentially improving outcomes. Further research on predictive biomarkers and immune microenvironment characteristics is crucial for personalizing immunotherapy approaches and enhancing their efficacy in managing recurrent ovarian cancer.

## 1. Introduction

Ovarian cancer is a leading cause of cancer-related deaths among women worldwide. Recurrent ovarian cancer remains a significant clinical challenge due to how difficult it is to treat and its high mortality rate [1]. Because resistance develops, traditional treatments like chemotherapy, surgery, and radiotherapy show limited efficacy in recurrent ovarian cancer. Therefore, efforts to reveal the mechanisms responsible for resistance to traditional treatments have increased significantly [2,3]. Simultaneously, new treatments have provided a new direction for improving the outcomes of recurrent ovarian cancer. Immunotherapy is emerging as an encouraging treatment for malignant melanoma, lung cancer, and renal cancer [4]. However, ovarian cancer’s response rate to immunotherapy is less than satisfactory [5].

The limited success of existing immunotherapies in ovarian cancer is due to several factors, including the tumor’s immune evasion strategies, such as the immunosuppressive tumor microenvironment, the presence of regulatory T cells (Tregs), and the macrophage [6]. A recent study showed that the chemotherapy in ovarian cancer induces myeloid-driven, spatially confined T cell exhaustion, where myeloid cells release immunosuppressive factors that impair T cell function within specific tumor regions, contributing to the immunuosuppressive microenvironment of ovarian cancer [7]. Additionally, ovarian cancer’s heterogeneity and the lack of effective tumor-specific antigens contribute to suboptimal immune responses [8]. New immunotherapeutic strategies, including dual immune checkpoint inhibitors, CAR T cell therapy, and cancer vaccines, are being explored to overcome these challenges. Combining immune checkpoint blockades with other treatments, such as chemotherapy or targeted therapies, may enhance efficacy.

## 2. Mechanisms of Immunotherapy and Different Subtypes of Immunotherapy in Ovarian Cancer

Tumor cells undergo apoptosis and release tumor-associated antigens (TAAs) after chemotherapy, radiotherapy, or targeted therapy. TAAs are taken up and processed by antigen-presenting cells like dendritic cells and presented to T cells. The T cells are activated by binding T cell receptors with the presented antigens combined with additional costimulatory signal stimulation. Activated T cells can release cytotoxins or pro-inflammatory cytokines, either directly killing tumor cells or inducing other immune cells to eradicate tumors [4]. Immunotherapy can be classified as active or passive according to whether the host immune system is mobilized to attack cancer cells actively [4,9].

Active immunotherapy stimulates the patient’s immune system to recognize and attack cancer cells [10]. This therapy encourages the body to generate a sustained immune response. Active immunotherapy uses the following treatments: (1) cancer vaccines (e.g., neoantigen vaccines), which can activate the body’s immune system to generate a specific response against cancer cells; (2) immune checkpoint inhibitors (e.g., PD-1/PD-L1 inhibitors), which can suppress the function of immune checkpoint molecules, thereby enhancing the immune system’s ability to attack cancer cells; (3) oncolytic virus therapy (e.g., talimogene laherparepvec [T-VEC]), which selectively infects and kills cancer cells, releases tumor antigens, and stimulates an immune response; and (4) cell therapy that utilizes the patient’s immune cells (e.g., T cells or natural killer cells), which are modified or enhanced and then reinfused into the patient to boost the immune system’s disease-fighting capability [11,12].

Passive immunotherapy involves the administration of immune components like antibodies or immune cells directly to the patient. This therapy does not rely on the patient’s immune system to generate a response but provides immediate immune effectors [10]. Passive immunotherapy includes the following interventions: (1) monoclonal antibodies (e.g., trastuzumab), which can directly inhibit tumor growth or mark cancer cells for destruction by the immune system after binding to specific targets on cancer cells; (2) CAR-T cells, which are extracted from the patient, genetically engineered to express CARs that recognize specific cancer antigens, and reinfused into the patient to target and kill cancer cells; and (3) the adoptive transfer of cells (e.g., tumor-infiltrating lymphocytes [TILs]). This involves collecting and using a patient’s immune cells after expanding them outside the patient and reinfusing them into the patient [13].

## 3. Immune Microenvironment (IME) of Recurrent Ovarian Cancer (ROC)

The IME of ROC plays a crucial role in tumor progression, response to treatment, and, ultimately, patient prognosis. Understanding the characteristics and dynamics of the IME is essential to developing effective therapies, including immunotherapies [14]. Ovarian cancer is characterized by a highly heterogeneous tumor microenvironment, comprising not only malignant cells but also a wide range of immune cells, stromal components, and soluble factors that interact in complex ways [14]. As ovarian cancer recurs, the tumor undergoes dynamic changes, leading to a more complex and often suppressive IME, which significantly influences treatment outcomes [15]. The characteristics of the IME differ between platinum-sensitive recurrent ovarian cancer (PSROC) and platinum-resistant recurrent ovarian cancer (PRROC), making these subtypes essential for guiding therapeutic strategies.

### 3.1. IME in PSROC

The immune landscape of PSROC is typically characterized by a higher frequency of TILs and a lower expression level of immunosuppressive molecules than PRROC [15]. In PSROC, tumors are more likely to maintain an immune-permissive environment that supports immune cell infiltration and activity, especially of cytotoxic T cells. High levels of CD8+ T cells, critical effectors of the antitumor immune response, are often observed in these tumors and are associated with better prognosis and longer progression-free survival (PFS) [16]. These tumors typically show higher levels of immune-related gene expression. This includes those involved in antigen presentation, interferon (IFN) signaling, and T cell activation. Tumor-infiltrating lymphocytes (TILs), particularly those with high functional activity, can sustain immune pressure on tumor cells, contributing to clinical sensitivity to platinum-based chemotherapy [17,18].

Despite a generally more favorable immune landscape in PSROC, immune suppression mechanisms, such as regulatory T cells (Tregs) and myeloid-derived suppressor cells (MDSCs), exist but are typically less prominent than those in PRROC [17,19]. These features suggest that PSROC might benefit more from immunotherapeutic approaches that boost existing immune responses. Additionally, PSROC often has a higher expression of programmed cell death ligand 1 (PD-L1) and an activated type I interferon response, which plays a role in antitumor immunity. The relatively high tumor mutational burden (TMB) observed in some PSROC cases may also enhance neoantigen formation, making these tumors more susceptible to immune checkpoint inhibitors and other forms of immunotherapy [20].

### 3.2. IME in Platinum-Resistant Recurrent Ovarian Cancer (PRROC)

In contrast, the immune microenvironment in PRROC is characterized by a more immunosuppressive profile, contributing to poor responses to both chemotherapy and immunotherapy [21]. PRROC tumors frequently exhibit lower levels of CD8+ T cells and reduced TILs, leading to an inability to mount effective antitumor immune responses. In addition to a decrease in effector T cells, there is an increase in immunosuppressive cells, such as Tregs, MDSCs, and tumor-associated macrophages (TAMs), which inhibit immune cell activation and contribute to the tumor’s ability to evade immune surveillance [15].

The expression of PD-L1 in PRROC is often low, and the IFN signaling pathway is less active, both of which contribute to the tumor’s immune escape mechanisms [17]. Moreover, lower TMB and fewer neoantigens are observed in PRROC, which decreases the potential for immune recognition and reduces the likelihood of effective responses to immune checkpoint blockade [22].

Additionally, PRROC tends to have a more hypoxic and fibrotic tumor microenvironment, which further contributes to immune suppression by hindering immune cell infiltration and promoting the survival of resistant tumor cells [23]. These factors make PRROC more challenging to treat with standard therapies, and new strategies that target the specific immune evasion mechanisms in these tumors are being explored.

## 4. Progress in Immunotherapy for Recurrent Ovarian Cancer

### 4.1. Monotherapy of Immune Checkpoint Inhibitors (ICIs) in Recurrent Ovarian Cancer

Immune checkpoint inhibitors (ICIs) exert antitumor effects by activating the body’s immune response. Inhibitors targeting immune checkpoints such as PD-1, PD-L1, and CTLA4 have demonstrated significant efficacy in various cancers. Multiple clinical studies have shown that the objective response rate (ORR) of monotherapy with PD-1/PD-L1 inhibitors in recurrent ovarian cancer ranges from 4% to 15%. Moreover, the most commonly reported immune-related side effects (irAEs) in ovarian cancer patients included fatigue, rash, and diarrhea, which were generally mild to moderate in severity [24]. However, in patients with PD-L1 expression ≥ 5%, the ORR for PD-L1 inhibitor atezolizumab monotherapy significantly increases to 22.2% [25]. However, due to the unique immune microenvironment and immunogenomic profile (e.g., mutations in *PIK3CA, PTEN, ARID1A,* and the *MAPK* pathway-related *KRAS* mutations), the patients with ovarian clear cell carcinoma benefit more significantly from ICIs [26]. Therefore, apart from ovarian clear cell carcinoma, the overall response rate of monotherapy with ICIs in recurrent ovarian cancer remains low. Consequently, exploring the combination of immunotherapy with other therapeutic approaches to enhance efficacy has become a new treatment strategy.

### 4.2. Dual ICIs

Due to the lower satisfaction with the effect of monotherapy of ICIs in recurrent ovarian cancer, dual immune checkpoint blockade, targeting both PD-1/PD-L1 and CTLA4 pathways, is gaining attention. This can be attributed to the positive results achieved using this combination in preclinical studies. Several clinical trials were performed to examine the efficacy of dual immune checkpoint inhibition in ovarian cancer. In a phase II trial, 100 patients with recurrent ovarian cancer were allocated randomly to receive either nivolumab alone or in combination with ipilimumab. The results showed that the combination treatment was more effective than nivolumab alone (ORR 31.4% vs. 12.2%) [27], with a near doubling of the median PFS (4 vs. 2). Another clinical trial explored the efficacy of durvalumab and tremelimumab in patients with recurrent ovarian cancer, and the preliminary results showed durable responses in a subset of patients, with a disease control rate (DCR) of approximately 31.6% [28]. Meanwhile, the JAVELIN Ovarian 200, a phase III trial, investigated avelumab in combination with ipilimumab and failed to meet its primary endpoint of improving PFS [29]. Despite the disappointing overall survival data in NCT2580058, biomarker analysis revealed that patients with high *PD-L1* expression or tumor mutation burden (TMB) might benefit more from dual ICI therapy [29]. However, combination ICI therapy is associated with higher rates of immune-related adverse events, which can be severe and require the discontinuation of treatment [27,28,29]. Balancing efficacy with tolerability remains a crucial concern. At the same time, some patients may resist ICIs through mechanisms such as the loss of antigen presentation or mutations in the interferon signaling pathway [30]. Combination strategies targeting other immune checkpoints (e.g., LAG-3, TIM-3) [31] or integrating ICIs with therapies such as radiotherapy or vaccines are being explored to overcome resistance [32,33].

### 4.3. ICIs Combined with Chemotherapy or Radiotherapy

Monotherapy with immunotherapy for recurrent ovarian cancer has not shown promising efficacy, which is possibly due to the immunosuppressive microenvironment of ovarian cancer. Studies suggest that chemotherapy can activate effector cells, inhibit immunosuppressive cells, or enhance immunogenicity, thereby improving lymphocyte infiltration and converting “cold tumors” into “hot tumors”, which may improve the efficacy of immunotherapy. In the phase III JAVELIN Ovarian 200 trial evaluating avelumab, a PD-L1 inhibitor, in combination with chemotherapy in patients with platinum-resistant or refractory ovarian cancer, the trial did not achieve its primary endpoint of improving overall survival [22]. Similarly, several clinical studies have demonstrated that the combination of immunotherapy with chemotherapy has not significantly improved PFS or overall survival (OS) in patients with recurrent ovarian cancer [5]. Nevertheless, Zsiros et al. found that the combination of cyclophosphamide with bevacizumab and pembrolizumab significantly increased the ORR in recurrent ovarian cancer, reaching 37.5%, with a 6-month PFS rate of 70%. Among the platinum-resistant recurrent population, the 6-month PFS rate reached 59% [34]. However, the combination of ICIs with chemotherapy led to more frequent or severe irAEs, such as hematologic toxicities, gastrointestinal events, and hepatic dysfunction [29].

In clinical practice, we have also confirmed the efficacy of immunotherapy in a patient with platinum-resistant recurrent high-grade serous ovarian cancer. After surgery and chemotherapy, the patient developed multiple liver metastases, vaginal stump tumors, and multiple lymph node metastases in the pelvis and abdomen six months later. After four cycles of pembrolizumab treatment, the tumors significantly shrank. However, the disease progressed after 11 cycles of pembrolizumab, and three additional cycles of pegylated liposomal doxorubicin were administered, resulting in a 50% reduction in pelvic tumors and a decrease in liver metastases from 70% to 30%. Successful debulking surgery was subsequently performed. Multiple immunohistochemical analyses showed high levels of NK cells and CD8+ T cell clusters in the tumor tissue. It was speculated that chemotherapy enhanced the infiltration of immune cells into the tumor tissue, thereby augmenting the efficacy of immunotherapy, which enabled the patient to benefit from the treatment [35].

Similarly, irradiated tumor cells can undergo immunogenic cell death, releasing large amounts of tumor antigens, thereby acting as an in situ vaccine. Radiation can also induce the reprogramming of the tumor microenvironment by upregulating cytokines and chemokines, promoting T cell infiltration and antitumor responses [36]. In patients with various solid tumors, including ovarian cancer, stereotactic body radiation therapy (SBRT) combined with pembrolizumab has demonstrated significant antitumor activity, particularly in some patients with prior treatment resistance [37]. The most severe adverse events, including grade 3 or higher toxicities, were observed in 6 out of 73 patients. These included severe immune-related side effects such as pneumonitis, colitis, and liver dysfunction [37]. There was reasonable safety, and no severe adverse events were observed. The NRG-GY003 trial investigated the combination of ipilimumab and nivolumab with radiotherapy in recurrent ovarian cancer. Results revealed an improved ORR compared to ICIs alone, indicating that radiotherapy may synergistically potentiate the effects of immunotherapy [38]. However, the efficacy of immunotherapy combined with chemoradiotherapy in ovarian cancer patients requires further exploration and validation.

### 4.4. ICIs Combined with Anti-Angiogenic Agents

The inhibition of the binding of vascular endothelial growth factor (VEGF) to its receptor could suppress the development of cancer by restraining tumor angiogenesis, characterized by aberrant and undifferentiated vessels, and by accumulating T cell infiltration into the tumor, stimulating tumor immune responses, and enhancing T cell-mediated tumor cell killing [39]. Simultaneously, immunotherapy can activate tumor-specific T cells and promote the secretion of IFN-γ, leading to vascular normalization or regression. Immunotherapy can also stimulate cancer patients to produce autoantibodies that inhibit angiogenesis. Therefore, immunotherapy and anti-angiogenic agents have a mutually promoting effect [5].

However, in the IMagyn050 clinical trial, adding atezolizumab to the chemotherapy and bevacizumab regimen did not prolong patients’ PFS, but in a subgroup analysis of clear cell carcinoma patients (n = 51), the addition of atezolizumab improved PFS (HR = 0.64, 95% CI [0.33, 1.24]) [40]. In a clinical study led by Chinese researchers focusing on recurrent/persistent ovarian clear cell carcinoma after platinum-based chemotherapy failure, the combination of bevacizumab and sintilimab showed encouraging results, with an ORR of 40.5%, a DCR of 73%, and a 12-month duration of response (DOR) rate of 68.6%. Patients who achieved an objective response demonstrated durable benefits [41]. However, in patients with rare ovarian malignancies such as low-grade serous carcinoma and steroid cell tumors, no significant difference in PFS was observed between the atezolizumab plus bevacizumab group and the placebo plus bevacizumab group [42].

In summary, while immunotherapy combined with anti-angiogenic agents has not achieved success in newly diagnosed advanced ovarian cancer, PSROC, or rare platinum-sensitive recurrent epithelial ovarian cancers, regardless of its use as maintenance therapy or without chemotherapy, has shown great potential in clear cell carcinoma. Further research is needed to explore this potential.

### 4.5. ICIs Combined with Poly (ADP-Ribose) Polymerase Inhibitors (PARPi)

PARPi inhibits poly (ADP-ribose) polymerase (PARP), leading to the impaired repair of DNA single-strand breaks and ultimately causing the accumulation of double-strand breaks in cancer cells, particularly in the context of homologous recombination repair (HRR) deficiency, such as in *BRCA1/2* mutations. The accumulation of DNA damage can result in recognizing intracellular DNA fragments as exogenous pathogens, activating the stimulator of interferon genes (STING) pathway, and triggering a type I interferon response, thereby promoting immune system activation [43].

Studies have shown that PARPi can upregulate PD-L1 expression on tumor cells and increase T cell infiltration, particularly CD8+ T cells, in the tumor microenvironment, thereby enhancing tumor sensitivity to immune checkpoint inhibitors. In the TOPACIO/KEYNOTE-162 clinical trial, the combination of niraparib and pembrolizumab achieved an ORR of 25% and a DCR of 68% in 60 patients with PRROC. Among the 11 patients with *BRCA* mutations, the ORR was 45%, and the DCR was 73% [44]. However, in PSROC patients, adding atezolizumab to carboplatin combined with niraparib maintenance therapy did not prolong PFS. *BRCA* mutation and *PD-L1* expression had no significant impact on PFS [45]. In another clinical trial, DUO-O was used to evaluate the efficacy of carboplatin and paclitaxel chemotherapy combined with bevacizumab/durvalumab and olaparib in ovarian cancer patients without *BRCA* mutations. The results showed that compared to the control group, the median PFS in the triplet therapy group was extended by approximately 5 months, with a 38% reduction in the risk of death. In patients with homologous recombination deficiency (HRD), the median PFS in the triplet therapy group was extended by 14.3 months compared to the chemotherapy plus the bevacizumab monotherapy group [46].

In summary, the DUO-O study demonstrated the efficacy of combining chemotherapy with anti-angiogenic agents, PARPi, and immunotherapy in ovarian cancer patients. However, the benefit of immunotherapy remains relatively limited, leaving much room for further exploration.

### 4.6. ICIs Combined with Antibody–Drug Conjugates (ADCs)

ADCs combine the targeting specificity of antibodies with the potent cytotoxic capability of small-molecule toxins. The antibody component can precisely recognize and bind to specific antigens on the surface of tumor cells, delivering chemotherapy drugs directly to the target cells, thereby reducing damage to normal tissues [47]. ADCs can overcome some resistance issues associated with traditional chemotherapy and may reduce resistance caused by target mutations to some extent [47].

Folate receptor alpha (FRα) is overexpressed in most ovarian cancers, with an overexpression rate of 80–96% in serous epithelial ovarian cancer. FRα overexpression is a poor prognostic factor for ovarian cancer chemotherapy [48]. Mirvetuximab soravtansine (MIRV) is an ADC-targeting FRα composed of a humanized FRα-binding monoclonal antibody conjugated to the cytotoxic maytansinoid DM4 via a cleavable disulfide bond, achieving targeted antitumor activity. The FDA has approved MIRV for the treatment of PRROC.

In the FORWARD I clinical trial, MIRV did not show the superior efficacy compared to chemotherapy in PRROC. However, in patients with high levels of FRα expression, MIRV was more effective than chemotherapy (ORR 24% vs. 10%), and the incidence of grade 3 or higher adverse events was lower than that of chemotherapy (25.1% vs. 44%) [49]. In the FORWARD II clinical trial, in 14 patients with high FRα expression and PRROC, the use of MIRV combined with pembrolizumab showed promising clinical efficacy, with an ORR of 43%, a median DOR of 6.9 months, a median PFS of 5.2 months, and no severe adverse events [50]. Therefore, this combination exhibits good tolerability and a significant clinical benefit in patients with recurrent ovarian cancer. Furthermore, as ADC development targeting new antigens progresses, combined strategies with immunotherapy are expected to constitue a new approach to overcoming recurrent ovarian cancer.

### 4.7. Application of Tumor Vaccines in Recurrent Ovarian Cancer

Tumor vaccines are immunotherapy designed to stimulate the immune system to recognize and attack cancer cells. Tumor vaccines in ovarian cancer include dendritic cells (DC), whole tumor cells, and peptide/protein vaccines [51]. The most widely studied is the DC vaccine, which is composed of dendritic cells with tumor antigens. These vaccines activate T cells, thereby targeting and eliminating ovarian cancer cells [52]. The therapeutic use of ovarian cancer vaccines has been investigated in various clinical trials. However, tumor vaccines alone have not shown significant clinical responses [53].

A meta-analysis that included 32 clinical studies since 2002, involving 426 patients with advanced or recurrent ovarian cancer, found that the ORR of tumor vaccines was 4% (95% CI 1–7), with a median PFS of 13 months (95% CI 8.5–16.3), and a median OS of 39 months (95% CI 31–49), with no severe adverse effects reported [54]. Although vaccines have shown considerable safety, improving their efficacy remains crucial. A phase I/II study of Vigil examining the efficacy of a GM-CSF-secreting autologous tumor cell vaccine demonstrated extended PFS in combination with durvalumab in recurrent ovarian cancer patients with PD-L1 expression, highlighting the synergistic potential of combining these modalities [55].

Many challenges exist in terms of improving the clinical efficacy of tumor vaccines, including the tumor microenvironment heterogeneity, the availability of autologous dendritic cells, the laboratory and technical conditions required for vaccine production, and the costs involved in vaccine preparation [56]. Further research is needed to identify the optimal patient population for vaccine therapy. The hope is that therapeutic tumor vaccines can stimulate sustained immune activation, thereby controlling the disease and improving outcomes, especially for patients who do not derive significant benefits from PARPi.

### 4.8. Application of Adoptive Cell Therapy (ACT) in Recurrent Ovarian Cancer

Adoptive cell therapy is also frequently discussed in tumor immunology research. It involves using autologous or allogeneic TILs or peripheral blood lymphocytes cultured and activated in vitro and reinfusing them to target and attack cancer cells [57]. In recent years, the application of TILs in recurrent solid tumors has achieved positive results, and the therapeutic effects of TILs in recurrent ovarian cancer have also drawn significant attention. Previous studies have shown that TIL infiltration, especially for T cells, positively correlates with better prognosis in ovarian cancer. This suggests that ovarian cancer may benefit from passive immune cell infusion therapy [58].

In patients with PRROC, after standard immune-depleting chemotherapy and TIL infusion followed by IL-2 maintenance therapy for five days, all patients achieved stable disease (SD) status for at least three months, with two patients maintaining SD for five months, and the treatment was well tolerated [59]. In patients with PSROC, patients received three TIL infusions two weeks after completing two to four cycles of chemotherapy, followed by two more cycles of chemotherapy. Preliminary results showed that 14 out of 16 patients completed the treatment, with an ORR of 86%, including three complete responses (CR), nine partial responses (PR), and two SD cases. The average PFS was 10.7 months, which was significantly longer than that for patients receiving the same chemotherapy regimen at the same treatment line [60]. Additionally, in this study, one patient achieved long-term CR. However, safety evaluations indicated that adding IFNα increased the risk of thrombocytopenia and neutropenia, while the treatment without IFNα showed reliable safety [60]. Nonetheless, the limited number of patients enrolled in these studies and the lack of control groups highlight the need for more high-quality clinical trials to confirm the efficacy of TILs in recurrent ovarian cancer.

## 5. Biomarkers for Predicting the Efficacy of Immunotherapy

Not only has monotherapy with immunotherapy for recurrent ovarian cancer shown limited efficacy, but the combination of immunotherapy with chemoradiotherapy, anti-angiogenic agents, and PARPi has also yielded suboptimal results. Therefore, identifying appropriate biomarkers to select patients most likely to benefit from immunotherapy has become critical. However, no standardized biomarker is available to select patients best suited for immunotherapy effectively. *PD-L1* expression is an excellent predictive indicator; in non-small-cell lung cancer, metastatic genitourinary tumors, gastric cancer, and cervical cancer, patients with high *PD-L1* expression derive more significant benefits from immunotherapy [5]. In addition, *PD-L1* expression can predict the response of ovarian cancer patients to pembrolizumab [26]. However, the criteria for determining PD-L1 positivity vary across different studies. Patients with microsatellite instability (MSI) due to defects in mismatch repair genes (*MLH1, PMS2, MSH2, MSH6*) may also benefit from immunotherapy [61]. However, unlike endometrial cancer, the incidence of MSI in ovarian cancer is relatively low. TMB has gradually become an essential biomarker for assessing the efficacy of immunotherapy, as tumors with high TMB are more likely to generate neoantigens, thereby enhancing the immune system’s ability to recognize tumors and improving the effectiveness of immunotherapy. However, the overall TMB level in ovarian cancer is relatively low, which poses challenges for its application as a predictive biomarker for immunotherapy in ovarian cancer [62].

Additionally, gene expression profiles (GEPs), indicating a T cell inflamed microenvironment, such as elevated interferon-γ signaling, are promising predictors of ICI response. Multi-gene panels are being developed for clinical application [63]. Lymphocytes in the tumor microenvironment play an important role in predicting immunotherapy efficacy. Studies have found that high densities of CD8+ T cells in ovarian cancer are associated with better immune responses and prognosis [64]. Moreover, a higher density of cytotoxic T cells and a lower presence of regulatory T cells or myeloid-derived suppressor cells (MDSCs) predict better outcomes [65]. Not only is the quantity of lymphocytes necessary, but their functional status is also crucial. Functionally active T cells typically express high cytokine levels, enabling them to kill tumor cells [64]. Moreover, emerging evidence links gut microbiota diversity and specific bacterial species (e.g., *Akkermansia muciniphila*) to enhanced ICI response. Modulating the microbiome may offer new therapeutic opportunities [66]. Besides that, circulating tumor DNA (ctDNA) and exosomes can also provide information on tumor characteristics and treatment response [66]. Despite significant progress, the clinical translation of biomarkers faces hurdles, including assay standardization, heterogeneity among cancer types, and dynamic changes in biomarker expression. Future research should focus on refining biomarker assays, validating findings in diverse patient populations, and integrating biomarkers into routine clinical workflows.

In summary, research on biomarkers for predicting the efficacy of immunotherapy is progressing rapidly. Identifying and validating these biomarkers will help to better select patients suitable for immunotherapy, improving treatment success rates and patients’ quality of life. However, further validation and standardization are needed for the clinical application of these biomarkers to ensure their reliability and reproducibility.

## 6. Conclusions

The treatment of recurrent ovarian cancer remains challenging, but immunotherapy has emerged as a promising approach, offering new hope for patients. Given the pronounced heterogeneity of ovarian cancer, immunotherapy outcomes can vary significantly among individuals [8]. To address this, preclinical models such as in vitro systems, animal models, and patient-derived xenografts have been developed to support precision medicine [67]. While these models have inherent limitations, ongoing optimization aims to enhance their clinical relevance in ovarian cancer research. Despite existing challenges in clinical application, the growing body of studies and clinical trials continues to advance the field (Table 1). In the future, with advancements in technology and a deeper understanding, it is expected that immunotherapy combined with radiotherapy/chemotherapy, immunotherapy combined with anti-angiogenic agents, immunotherapy combined with PARPi, and immunotherapy combined with ADCs will play increasingly important roles in the treatment of recurrent ovarian cancer.

## Figures and Tables

**Table 1:** Ongoing clinical trials of immunotherapy in recurrent ovarian cancer.

Type of Treatment	Clinical Trial ID	Target Patients	Phase	Study Design	Status
Envafolimab + Lenvatinib + chemotherapy	NCT05422183	Recurrent platinum-resistant ovarian cancer	II	Envafolimab + lenvatinib + VP-16	Unkown
Immune checkpoint blockade + intraperitoneal chemo-immunotherapy	NCT03734692	Recurrent ovarian cancer	II	Intraperitoneal chemo-immunotherapy of (cisplatin + rintatolimod (TLR-3 agonist)) + intravenous pembrolizumab	Recruiting
Cryosurgery + NK immunotherapy	NCT02849353	Recurrent ovarian cancer	I and II	Cryosurgery + NK immunotherapy	Completed
Innocell autologous cellular immunotherapy	NCT06366490	Recurrent epithelial ovarian cancer	I	Innocell vaccine	Not yet recruiting
DCVAC/OvCa + chemotherapy	NCT02107378	Platinum resistant epithelial ovarian canrcinoma	II	DCVAC/OvCa + chemotherapy	Terminated
Adebrelimab + non-platinum chemotherapy + fuzuloparib	NCT06600841	Recurrent platinum-resistant ovarian cancer	II	Adebrelimab + Non-platinum chemotherapy + Fuzuloparib	Not yet recruiting
Adoptive T cell therapy	NCT04072263	Recurrent ovarian cancer	I and II	Carboplatin-paclitaxel + Tumor Infiltrating Lymphocytes	Unknown status
Chemotherapy + Peg-intron and p53 SLP vaccination	NCT01639885	Platinum resistant ovarian cancer	I and II	Gemcitabine + Pegylated Interferon Alpha-2b (Peg-Intron) +/− p53 Synthetic Long Peptide (p53 SLP) Vaccine	Completed
Envafolimab + lenvatinib + chemotherapy	NCT05422183	Recurrent platinum-resistant ovarian cancer	II	Envafolimab + lenvatinib + VP-16	Unkown
Immune checkpoint blockade + intraperitoneal chemo-immunotherapy	NCT03734692	Recurrent ovarian cancer	II	Intraperitoneal chemo-immunotherapy of (cisplatin + rintatolimod (TLR-3 agonist)) + intravenous pembrolizumab	Recruiting

The latest clinical trial information was updated on the Clinical Trials website as of 21 December 2024.

## Data Availability

Not applicable.
